# Safety Mechanisms and Risk Mitigation in Generative AI Mental Health Chatbots: A Systematic Scoping Review

**DOI:** 10.3390/healthcare14101395

**Published:** 2026-05-20

**Authors:** Lotenna Olisaeloka, Chris G. Richardson, Angel Y. Wang, Richard J. Munthali, Daniel V. Vigo

**Affiliations:** 1 Department of Psychiatry, Faculty of Medicine, University of British Columbia, Vancouver, BC V6T 0A6, Canada; 2 School of Population and Public Health, Faculty of Medicine, University of British Columbia, Vancouver, BC V6T 1Z8, Canada

**Keywords:** artificial intelligence, generative AI, AI chatbots, mental health chatbots, AI safety, ChatGPT

## Abstract

Background: Generative AI (GenAI) mental health chatbots are increasingly being developed to help address persistent barriers to mental healthcare. Unlike earlier rule-based and retrieval-based systems, GenAI chatbots generate open-ended outputs that can be inaccurate and unsafe. Documented harms from general-purpose GenAI chatbots have highlighted the need for purpose-built interventions with dedicated safeguards, yet how safety is implemented in such interventions remains poorly understood. Methods: This scoping review followed the Joanna Briggs Institute methodology and PRISMA-ScR guidelines, with a prospectively registered and peer-reviewed protocol. A systematic search of seven academic databases and search engines including MEDLINE, Scopus, PsycINFO, ACM Digital Library, IEEE Xplore, Google Scholar and Consensus was conducted in July 2025. Two reviewers independently screened records and extracted data. Safety mechanisms and risk mitigation strategies were narratively synthesised across three pre-specified domains: technical safeguards, pre-deployment safety considerations, and delivery-phase risk mitigation strategies. Results: Twenty-one studies across 11 countries were included. Most interventions incorporated at least one technical safety mechanism, most commonly fine-tuning and prompt engineering. A smaller subset implemented layered safety architectures combining retrieval systems, content filters or risk classifiers, and rule-based algorithms. Pre-deployment safeguards included clinical expert and user co-design approaches, research ethics procedures, and data privacy measures. During intervention delivery, detailed onboarding with role clarification was common, but human oversight was limited. Crisis referral protocols varied in rigour but were mostly underdeveloped, and systematic adverse event monitoring was sparse. Documented safety failures included missed suicidal ideation and provision of inaccurate clinical information. Conclusions: GenAI chatbot interventions require a robust sociotechnical approach that integrates technical safeguards with user co-design, procedural controls, and human oversight. Future research is needed to evaluate efficacy, improve safeguards and standardise safety outcome measurement. Regulatory oversight proportional to the risks these systems carry is required to enable integration into stepped or blended mental healthcare.

## 1. Background

One in four people globally will experience a mental disorder in their lifetime, yet access to treatment remains severely constrained [1]. Workforce shortages, high costs, geographic distance, and stigma mean that up to 85% of people especially in low- and middle-income countries receive no care at all [2,3]. This treatment gap has driven sustained interest in digital mental health interventions (DMHI), with chatbots among the most widely explored delivery tools [4].

Conversational agents (CA), commonly called chatbots, are not new to digital mental health. In fact, the first chatbot named ELIZA developed in 1966 by Joseph Weizenbaum was programmed to be a Rogerian therapist [5]. Over the last two decades, rule-based and retrieval-based chatbots have been used to deliver psychotherapeutic interventions, with reviews documenting their feasibility and modest efficacy across various conditions including depression, anxiety, and substance use [6,7,8,9,10]. The safety of these earlier generations of chatbot interventions, however, received comparatively limited research attention, with few studies evaluating safety as a primary outcome [11]. This is largely because they were explicitly programmed to deliver pre-scripted content with little room for unpredictable and potentially harmful outputs [11,12]. Thus, the technical risk profile of these systems was mostly manageable through careful content design. However, the development of large language model (LLM)-based chatbots has fundamentally changed this status quo.

The intrinsic characteristics of generative artificial intelligence (GenAI) introduce novel safety risks in chatbot interventions. LLMs generate responses probabilistically from learned patterns across vast training data, meaning their outputs are open-ended and cannot be fully anticipated during intervention design [13,14]. This creates a unique safety problem, one that content curation alone cannot solve. At the same time, these systems offer capabilities that earlier chatbots could not including contextually sensitive, empathetic, and personalised responses that more closely approximate the relational qualities of human-delivered support. Emerging research suggests that these qualities contribute to improved user engagement and efficacy [15,16].

In the absence of adequate access to mental health care, many have already turned to general-purpose LLM chatbots such as ChatGPT (by OpenAI) and Claude (by Anthropic) for support. Research suggests that therapy and companionship are among the most widely reported uses of GenAI [17]. A nationally representative US survey found that up to one in five adolescents and young adults had used GenAI tools for mental health advice. A separate survey of US adults with self-reported mental health conditions found that nearly half of those who used LLMs had done so specifically for psychological support [18]. These findings possibly reflect the low cost, immediate availability, personalised support and stigma-free nature of GenAI-based interactions, particularly among people who cannot access conventional care.

General-purpose LLMs were not designed for mental health use, and evidence of harm has accumulated alongside their growing adoption. These models are prone to hallucination, producing confident-sounding but factually incorrect content [19]. They also tend toward sycophancy, prioritising the generation of agreeable and validating responses above nuanced and clinically appropriate ones. In mental health contexts, these properties carry serious consequences. Interactions with AI chatbots have been linked to adverse outcomes including psychosis and suicide [20,21].

Ethical and safety concerns regarding general-purpose chatbots make the case for purpose-built GenAI mental health chatbot interventions that can facilitate user safety. User safety in digital mental health refers to protecting individuals from psychological, physical, and informational harm, safeguarding privacy and overall wellbeing, and mitigating risks associated with use [12]. Such safety considerations could be implemented across the intervention lifecycle, from how the underlying model is trained and constrained to how users are supported during deployment including crisis identification and escalation. Unlike general-purpose chatbots, purpose-built interventions can incorporate safety mechanisms such as constrained model behaviour, expert-validated training data, crisis detection pipelines, and human oversight as core design features. However, how these safety considerations are actually operationalised across purpose-built GenAI mental health chatbot interventions remains fragmented and poorly understood. Existing reviews of digital mental health chatbots have focused largely on efficacy and have mostly included non-generative systems, meaning their findings offer limited insight into how safety is operationalised in LLM-based interventions specifically [6,8,9,11,15].

At the same time, regulatory scrutiny of AI in mental health is accelerating. The US Food and Drug Administration (FDA) through its Digital Health Advisory Committee is considering oversight approaches to generative AI-enabled mental health tools [22]. Several US states have also passed laws regulating chatbot use in clinical contexts [23]. In the European Union, the AI Act establishes a risk-based framework where high-risk AI systems such as those used in healthcare must meet strict requirements for transparency and human oversight [24]. This regulatory momentum reflects a shared recognition that the field needs clear standards. Yet, those standards cannot be developed without a systematic understanding of what safety currently looks like in practice. This review addresses that gap by systematically mapping the safety mechanisms and risk mitigation strategies reported across existing GenAI mental health chatbot interventions. It is guided by the following research questions:

1. What technical safety mechanisms are integrated into generative AI mental health chatbot interventions during design and development?
2. What procedural and governance-level risk mitigation strategies are implemented during the deployment of these interventions?
3. What adverse outcomes are being tracked and how are they monitored and reported?

## 2. Methods

This scoping review followed the Joanna Briggs Institute (JBI) methodology for scoping reviews [25] and adhered to the Preferred Reporting Items for Systematic Reviews and Meta-Analyses extension for Scoping Reviews (PRISMA-ScR) [26]. The review protocol was prospectively registered on the Open Science Framework (https://doi.org/10.17605/OSF.IO/HSNXA, accessed on 26 August 2024) and subsequently published following peer review [12]. Review findings are reported across two companion papers. This paper focuses on safety mechanisms and risk mitigation strategies; intervention design and user experience findings are reported separately [27].

### 2.1. Search Strategy

Seven academic databases and research search engines were searched, comprising MEDLINE (Ovid), Scopus, PsycINFO, ACM Digital Library, IEEE Xplore, Google Scholar, and Consensus. The search was initially conducted in July 2024 and updated in July 2025. Search terms combined Medical Subject Headings (MeSH) and keywords across four conceptual domains: generative artificial intelligence and large language models, conversational agents and chatbots, mental and substance use disorders, and psychological therapies and interventions. The full search strategy is detailed in Appendix A. Retrieved records were deduplicated and managed in Covidence [28]. Two reviewers independently screened titles and abstracts followed by full texts. Disagreements were resolved through discussion, with a third reviewer available for adjudication.

### 2.2. Eligibility Criteria

Eligibility was determined using the Population, Concept, Context (PCC) framework as specified in the published protocol [12]. Primary peer-reviewed studies were eligible if they reported on the design, deployment, or implementation of a GenAI mental health chatbot intervention and included safety-relevant information such as technical safeguards, risk mitigation strategies, and adverse events. No restrictions were placed on population, age, gender, clinical diagnosis, or geographic setting. Non-generative interventions, secondary literature, and non-English language articles were excluded. The restriction to English-language publications may introduce language bias and is acknowledged as a limitation.

### 2.3. Data Charting and Synthesis

A data extraction form was developed and piloted a priori as described in the published protocol. Safety-relevant extraction variables were organised around three pre-specified domains: (i) AI/ML-based technical safety mechanisms such as fine-tuning and retrieval-augmented generation (RAG); (ii) pre-deployment safeguards such as ethical and data privacy procedures; (iii) deployment-phase risk mitigation strategies such as human oversight and crisis identification protocols. Extraction was completed by two reviewers via Covidence, with discrepancies resolved by consensus. Findings were narratively synthesised and organised thematically, guided by the research questions.

## 3. Results

**Figure 1:**
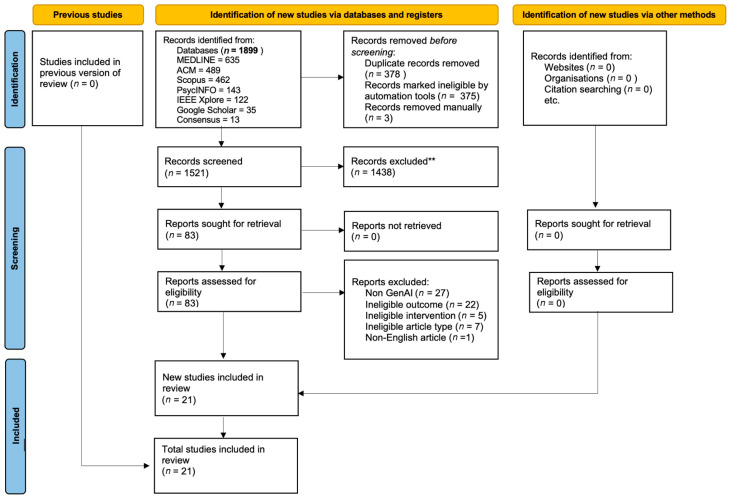
PRISMA diagram illustrating the search strategy implementation and results of the study selection process.

The systematic search yielded 1899 records, of which 21 studies met the eligibility criteria following title and abstract screening and full-text review. The PRISMA flow diagram is presented in Figure 1 and the reporting checklist is included in the Supplementary files. The included studies were conducted across eleven countries between 2023 and 2025, spanning prototype evaluations, feasibility studies, mixed-methods evaluations, randomised controlled trials, and one real-world implementation study. A summary of the included studies is presented in Table 1 [27].

The 21 included interventions were predominantly CBT-based and delivered through text-based, non-embodied chatbots via mobile applications or web-based platforms. A small number utilised voice, avatar-based, gamified, or augmented reality interfaces to enhance engagement and realism. Target conditions spanned depression, anxiety, post-traumatic stress disorder, eating disorders, and dementia, with most interventions designed for adult general or clinical populations. Some recruited more specific groups including university students, first responders, older adults with dementia, and their caregivers. Full details of intervention design characteristics and user experience outcomes are reported in Olisaeloka et al. (2026) [27].

### 3.1. Safety of Generative AI Mental Health Chatbot Interventions

**Figure 2:**
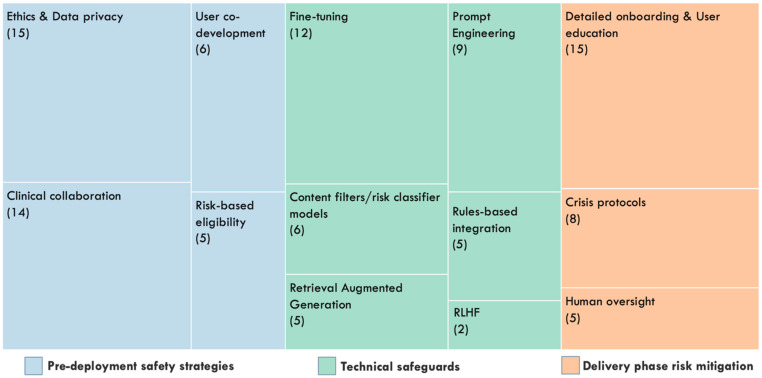
Types and frequencies of safety mechanisms and risk mitigation strategies implemented in the included generative AI mental health chatbot interventions (*n* = 21). The tree map visualises the relative frequency with which different safety and risk mitigation strategies were reported across included studies. Numbers in brackets indicate the number of studies that reported implementing each strategy. Individual interventions frequently implemented multiple safety strategies; therefore, categories are not mutually exclusive, and totals exceed the number of included studies.

Safety considerations were reported across most included studies, though the depth and explicitness of reporting varied considerably. Safety and risk mitigation strategies were mostly aimed at ensuring the correctness and appropriateness of generated responses, maintaining research ethics, protecting confidentiality and data privacy, and detecting and managing crises. These strategies were thematically mapped into three broad domains: technical (AI/ML) safety mechanisms, pre-deployment safeguards, and delivery-phase risk mitigation approaches. Figure 2 summarises the different types and frequencies of these safety strategies across included studies.

### 3.2. Technical (AI/ML) Safety Mechanisms

Safety was often embedded into the underlying AI systems through choices about training data, chatbot architecture, retrieval methods, and AI/ML risk detection modules. These technical measures aimed to reduce hallucination, restrict conversations to a predefined therapeutic scope, and flag risks in real time. The most commonly reported technical measure was fine-tuning on curated datasets (*n* = 12), often implemented alongside prompt engineering (*n* = 9). Other technical approaches included retrieval-augmented generation (RAG), layered content filtering and risk classification models, and hybrid architectures integrating generative and rule-based components (Figure 2).

#### 3.2.1. Fine-Tuning

Rather than relying on general-purpose LLMs, developers trained or adapted base models on domain-specific datasets to improve clinical relevance. The source and curation of training data varied considerably. Several conversational systems were fine-tuned on curated psychological or therapeutic data [16,29,35,38,39,41,42,47,48,49], although two studies relied on web-scraped and synthetic datasets [31,40].

The training datasets mostly featured structured dialogues from counselling or therapy sessions. Therabot [16] comprised a transformer-based LLM ensemble (Falcon-7B and LLaMA 2-70B) fine-tuned via quantised low-rank adaptation (QLoRA) on expert-curated transcripts from CBT sessions. GenAI Woebot was built on the Ada-002 model trained on a proprietary catalogue of prior user conversations and FAQs to ground the CA in an established knowledge base [29].

The creators of Clare R indicated that multiple fine-tuned models were developed through a collaborative process involving psychologists and conversational AI specialists to ensure therapeutic alignment and adherence to ethical guidelines [42]. Likewise, DrBot [47] developers fine-tuned DialoGPT on 7000 lines of therapy session transcripts and then selected the best-performing fine-tuned model for deployment. TeaBot’s [38] GPT-3 models were trained on 240 cognitive distortion examples sourced from CBT literature by psychology students and validated by a licensed psychologist. VCounselor [48] was a lightweight LLM (ChatGLM2-6B) trained on 80 annotated counselling cases to minimise misinformation and align outputs with DSM 5 diagnostic frameworks.

Other interventions leveraged local/culturally aligned datasets. Psy-LLM [49] was pre-trained on two Chinese LLMs (PanGu and WenZhong), and then fine-tuned on web-crawled Chinese mental health content and a 56,000–question–answer pair dataset verified by psychologists. *Emohaa* [41] was fine-tuned on an emotional support dataset, constructed using trained human supporters and then translated into Chinese. Similarly, the Carebot [39] emotion detection model was fine-tuned on five annotated datasets, and further adapted to the local language and Malaysian cultural context. In contrast, Wellness Buddy [40] intended for Kenyan students used non-adapted, publicly available mental health conversational datasets, with additional synthetic data to cover more user intents and responses.

#### 3.2.2. Prompt Engineering

Prompt engineering was explicitly reported in nine studies, functioning either as the primary technical safeguard or in combination with fine-tuning and RAG [16,29,31,33,36,37,45,46,47].

Several systems used prompts to define therapeutic personas and constrain conversational tone and scope. For instance, the anxiety intervention by Manole et al. [37] used ChatGPT with prompt engineering grounded in a clinically validated assessment tool, so that the chatbot’s questions and feedback followed an established structure. Similarly, HopeBot [31] relied on GPT 3.5 Turbo without specific fine-tuning, but used structured prompts to enforce PHQ-9 logic for depression screening and to avoid inappropriate responses. Prompts specified question order, scoring rules, and discouraged deviation from the screening script.

In ArtTheraCat [45], prompts instructed the model to act as a supportive art therapist, manage conversation flow, track image history, and avoid creating distressing images. MindTalker [46] used GPT-4 with prompts to align its responses with reminiscence therapy. ExpandXR’s [33] AI characters were defined through persona prompts that specified dialogue style and constrained behavioural patterns during augmented reality exposure tasks.

Other studies used prompting to ensure relevance and response accuracy. Therabot [16] was prompted to use evidence-based strategies and conversation history to generate responses. ComPeer [36] employed a chain of thought (CoT) prompting, combined with a memory module, to encourage context-aware responses and reduce hallucinations. DrBot prompted GPT 3.5 to refine responses and limit harmful content [47]. Even systems that otherwise relied on RAG sometimes used prompts as an additional safety layer. Ana [30], for instance, used prompting to guide how retrieved content could be expanded or rephrased by the GPT 3.5 model.

#### 3.2.3. Retrieval Augmented Generation (RAG)

Several chatbots combined LLMs with retrieval systems drawing on curated knowledge bases to reduce hallucinations and maintain alignment with validated information [29,30,38,48]. VCounselor [48] incorporated a knowledge retrieval system using structured DSM-5 data. User utterances were mapped to diagnostic frameworks and relevant knowledge snippets retrieved to guide the LLM’s responses. GenAI Woebot embedded the fine-tuned LLM inside a content management system that relied on retrieval from a proprietary psychoeducational library. The conversational system retrieves answers from the curated library, with the fine-tuned LLM filling gaps only when the knowledge base is insufficient to directly answer a user’s query. However, all responses were filtered so that participants never interacted with completely unconstrained model outputs [29]. Ana [30] used GPT 3.5 for question answering with a retrieval step from an expert-curated knowledge base. It adopted a thresholding mechanism that retrieved and rephrased verified content when available while still enabling flexible conversation. TeaBot [38] also used retrieval to support Socratic questioning by combining a fine-tuned classifier with RAG to select relevant content for cognitive restructuring. Unlike in Ana, the retrieval system was linked with an uncertainty response that allowed the model to abstain when the classification threshold confidence was low. Taken together, these systems illustrate patterns where generative models are wrapped in RAG pipelines that pull from curated libraries to reduce hallucination and ensure alignment with verified therapeutic content.

#### 3.2.4. Filtering Systems, Hybrid Architectures and Reinforcement Learning

A subset of interventions moved beyond standalone LLMs and instead embedded the generative component within layered architectures often combining content filters, risk classifiers, and rule-based logic [16,29,32,40,41]. These systems reflected the most deliberate safety engineering among the included studies.

Limbic Care [32] integrated GPT-4 within a proprietary “limbic layer”, a set of ML modules that interpreted user inputs, detected emotional states, and supported decision-making and conversation safety. Clare R [42] utilised a similar approach by combining a fine-tuned LLM with a separate ML model that filtered both inputs and outputs while intentionally “over flagging” potentially harmful content for human review.

Content filters and risk classifiers were deployed in several studies for crisis and self-harm detection. Therabot [16] combined its fine-tuned LLMs with a crisis detection model that screened messages for crisis-related language. Two other studies utilised the same approach for suicidality and self-harm risk detection [41,44]. For the non-fine-tuned model by Vossen et al. [44], when such topics were detected, the system bypassed ChatGPT entirely and served rule-based responses that signposted crisis lines.

Several systems constrained generative components within rule-based therapeutic workflows [29,32,37,42,44]. For example, Clare [42] and GenAI Woebot [29] used rule-based matching to map user inputs to predefined CBT exercises or FAQ content, with the LLM providing empathic, natural language interactions around fixed therapeutic content or self-help exercises. The personal AI assistant in Vossen et al.’s study [44] relied on Rasa for intent recognition and detection of high-risk topics, while reserving the ChatGPT API for lower-risk, supportive conversations.

Woebot [29] demonstrated the most fully articulated safety pipeline. All user messages first passed through a proprietary NLP-based content classifier serving as a “safety net to filter inappropriate requests”. A RAG layer then retrieved responses from Woebot’s curated library and handed the query along with additional safety prompting to a fine-tuned LLM for response generation. The system also included an ensemble of classification models that filtered hate, sexual violence, and self-harm language as a final output validation step before presenting it to the user. Thus, participants never interacted with raw LLM outputs at any stage.

A small number of studies implemented adaptive loops or reinforcement learning from human feedback (RLHF) which allowed users to confirm or correct emotional state classifications and thereby reduce the risk of irrelevant and potentially unsafe misinterpretations [33,37].

### 3.3. Pre-Deployment Safety Considerations

#### 3.3.1. Expert and User Co-Design

Clinical and domain expert involvement in intervention design was reported across most included studies as a key safety measure. Their contributions primarily included curation and validation of training datasets, input on ethical design, and preliminary evaluation of chatbot responses [16,29,30,32,33,38,39,41,42,44,46,47,49]. For example, psychologists helped design Clare R’s hybrid chatbot architecture and curated the training examples [42]. Therabot’s training data was also curated by a board-certified psychiatrist and clinical psychologist to ensure clinical alignment [16]. MindTalker underwent iterative co-design with experts (human–computer interaction researchers, dementia experts and therapists) and end-users, with the final version selected after eleven iterations based on UX and safety considerations [46].

A small number of studies conducted formal pre-deployment performance testing. Woebot developers carried out a readiness assessment using up to 42 different user personas, with a defined pass criterion specifying that generated outputs must not provide clinical advice, diagnoses, or offensive language. No violations were observed across this evaluation [29]. Lai et al. [49] had academic experts conduct a detailed evaluation of cleaned training datasets for accuracy, relevance, and coherence, alongside human validation of AI-generated responses before deployment. Mental health professionals also evaluated DrBot and Limbic before deployment and user evaluation [32,47]. In the latter, clinicians rated the system with the limbic layer higher on therapeutic alliance and overall performance in the preliminary evaluation.

End-user involvement in pre-deployment design was less consistent but present in a few interventions, mostly to explore acceptability, usability and safety risks. Ana [30] was informed by semi-structured interviews with caregivers which shaped acceptable features and boundaries. The personal AI assistant underwent co-design with students and medical experts before broader testing [44]. ComPeer [36] iteratively incorporated participant feedback into design refinements. ExpandXR [33] was co-developed with veterans and clinicians to ensure realism without inducing undue distress.

#### 3.3.2. Research Ethics and Data Security

Most empirical evaluations reported institutional ethics approval and standard confidentiality procedures. Several studies noted risk-based eligibility criteria, informed consent, data de-identification and secure storage of interaction logs. Five studies excluded participants based on suicidal ideation [16,29,39,41,42], with Heinz et al. [16] additionally excluding people with severe depression, mania and psychosis, and Sabour et al. [41] also excluding those in therapy or on psychiatric medication. Ng et al. [39] excluded users with severe depression or active suicidal ideation using PHQ-9 screening prior to access. These eligibility decisions functioned as pre-deployment risk management strategies, limiting exposure of the most vulnerable users.

Data security approaches were reported in the majority of studies but varied in their rigour. The Wellness Buddy intervention proposed a robust data privacy approach involving local data storage on the user’s device, with only the user able to access it [40]. Leora [43] proposed a comprehensive privacy architecture including data encryption and stated compliance with national privacy law. Two studies also reported on users’ rights to access and delete their data [36,43]. However, data security measures were not uniformly reported, and some users expressed concerns about data privacy.

### 3.4. Intervention Risk Mitigation and Adverse Outcomes

#### 3.4.1. Onboarding, User Education, and Role Clarity

Many interventions attempted to reduce safety risks at the point of entry by clarifying the chatbot’s capabilities and limitations. Several explicitly stated that the chatbot was not a replacement for professional therapy and encouraged users to seek human support for severe problems or crises [29,35,39,42,43,48,49]. Clare R and Woebot used structured onboarding to explain functionalities and privacy policies, set boundaries for interaction, and educate users about appropriate use [29,42]. Vossen et al. [44] conducted a dedicated 30 min introductory video call with each participant before the intervention began, delineating the study scope and chatbot functionality in detail. MindTalker [46] users were advised to complete onboarding with a family member or caregiver present, a co-use design feature particularly relevant for its dementia population. Several studies also preserved user agency during the intervention itself. For instance, ArtTheraCat allowed users to manually terminate sessions at any point, triggering a defined closure protocol with a final supportive message and session summary [45].

#### 3.4.2. Human Oversight and Clinical Integration

Only a few interventions mitigated risk by maintaining ongoing human oversight [16,41,42,48] or embedding the chatbot within clinical services [32]. *Limbic Care* was integrated into routine group CBT within the NHS, with clinician supervision and constant monitoring of conversations for harmful responses [32]. Clare and Emohaa had human moderators monitor conversations and review flagged content [41,42]. Therabot implemented the most rigorous human-in-the-loop (HITL) approach in which all chatbot responses were supervised by clinicians, who intervened when the bot produced inappropriate content such as medical advice [16]. VCounselor [48] also maintained real-time supervision of AI-generated responses by trained counsellors throughout the sessions. ExpandXR [33] was entirely clinician-controlled, with therapists observing AI character behaviour throughout and retaining authority to adjust exposure intensity or terminate the scenario at any point. ComPeer [36] maintained ongoing contact between the research team and participants throughout the intervention period, representing a lighter but nonetheless present form of human oversight. Most other studies reported no human monitoring during intervention delivery.

#### 3.4.3. Crisis Response Protocols

Crisis response protocols were present across several interventions but varied substantially in their robustness. The most common approach was directing users in crisis to external emergency services and crisis helplines. Woebot [29] used NLP-based risk detection to flag concerning content and route users to helplines and crisis resources. Clare R’s safety framework went further by also blocking further app use in addition to redirecting users to crisis services when safety concerns were detected. Emohaa [41] triggered a similarly defined crisis management protocol when its suicide risk classification model detected concerning content, ensuring detection was linked to a response pathway. In contrast, several studies reported no detailed crisis protocol, providing only general instructions to seek external help for emergencies [37,38,47].

#### 3.4.4. Adverse Event Monitoring

Systematic adverse event monitoring during deployment was rare and often sparsely described. Notably, no study provided an explicit definition of what constituted an adverse event in the context of a GenAI chatbot intervention, making it difficult to assess the scope and consistency of monitoring across studies. Only two studies reported formal adverse event monitoring processes. Woebot’s trial included safety and adverse event monitoring through both in-app exchanges and session notes, with each potential event recorded. The authors documented four instances of undesired behaviour including one message endorsing being hungover after drinking, but no serious harms [29]. Heinz et al. [16] reported adverse events such as inappropriate medical advice, which required direct human intervention on thirteen occasions to provide correction and safety guidance. Most other interventions did not include systematic, standardised reporting of adverse events and potential harms. However, critical safety issues were raised in some instances. HopeBot failed to identify and respond appropriately to simulated suicidal messages, providing generic assurances rather than directing users to emergency care [31]. In Liu et al. [35], the chatbot provided inaccurate information such as citing non-existent articles.

**Figure 3:**
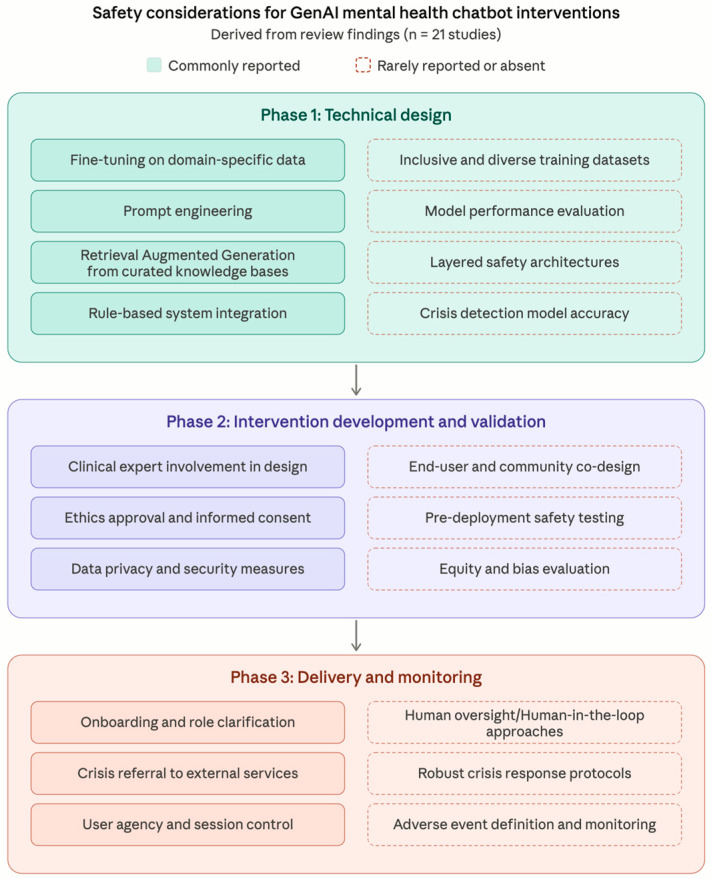
Lifecycle framework of safety design, evaluation and reporting considerations for generative AI mental health chatbot interventions. The framework maps commonly reported safety practices (solid borders) alongside identified gaps (dashed borders) across three phases: technical design, intervention development and validation, and delivery and monitoring. Items on the left reflect practices reported across multiple included studies; items on the right represent areas that were rarely addressed or absent.

Taken together, the safety practices and gaps identified across included studies reveal a pattern in which technical safeguards were most commonly addressed while delivery-phase protections, particularly human oversight, crisis response, and adverse event monitoring, were least developed. Figure 3 organises these findings into a lifecycle framework, mapping commonly reported practices alongside identified gaps across three phases of intervention development and deployment. The framework is grounded in findings from the existing literature and is intended to inform future work on safety planning, evaluation and reporting.

## 4. Discussion

This scoping review systematically mapped safety mechanisms and risk mitigation strategies across 21 purpose-built GenAI mental health chatbot interventions. The evidence base is nascent, with most included studies at early development or feasibility stages and only a small number of efficacy trials. Safety was addressed inconsistently across included studies, with a small subset indicating that layered technical safeguards, combined with robust crisis protocols and human oversight, represent emerging best practice. Most systems, however, relied on minimal or poorly specified safeguards, and the near absence of systematic adverse event monitoring means the true extent of harm from these systems remains unclear.

Review findings suggest that safety in GenAI mental health chatbot interventions is best understood as a complex sociotechnical problem rather than as a feature of the conversational agent alone. While prompt engineering and fine-tuning on curated clinical datasets is an important foundation, the probabilistic and open-ended nature of LLM outputs means that such safeguards can be bypassed by inputs the model was not trained to anticipate. Studies with the most credible safety profiles were those that integrated multiple safety measures, combining technical safeguards with crisis management protocols and human-in-the-loop pathways [16,29,42]. Such comprehensive approaches appeared to consistently outperform simpler safety architectures. Interventions relying primarily on prompt engineering without complementary safeguards showed the most visible failures, including missed suicidal content and provision of inaccurate clinical information. This finding is consistent with external evaluations of widely used mental health chatbots, which found that safety-oriented prompting and fine-tuning frequently failed to prevent crisis response failures and over-validation of harmful inputs [50,51]. These safety failures are particularly consequential given the known tendency of LLMs toward hallucination and sycophancy which can reinforce anthropomorphisation, trigger delusional thinking patterns, or delay appropriate crisis intervention [14,19,20]. These patterns further reflect a broader AI safety proposition that risks in AI systems arise not from algorithms in isolation but from the interaction between model behaviour, deployment context, and governance arrangements [52].

The lifecycle framework presented in Figure 3 maps the safety practices reported across included studies alongside key gaps identified within each phase of intervention design and delivery. Technical design considerations were commonly addressed, with fine-tuning, prompt engineering, and retrieval-augmented generation reported across most included studies. Still, critical technical aspects such as diversity of training data, model performance evaluation, algorithmic bias detection, and accuracy of crisis models remained largely unexplored or unreported prior to deployment. Intervention development and validation practices such as expert co-design and ethics procedures were frequently described, although pre-deployment safety testing and equity evaluation were rare. The most critical gaps clustered in the delivery and monitoring phase, where human oversight, robust crisis response protocols, and adverse event monitoring were insufficient or largely absent. This pattern suggests that current development efforts prioritise constraining model behaviour at the architectural level while under-investing in human-centred design, and the procedural mechanisms needed to mitigate harms once interventions reach users. To develop ethical and safe GenAI-based interventions, addressing technical design alone is insufficient without corresponding investment in equitable co-design, governance protocols, and ongoing monitoring throughout the intervention lifecycle.

Crisis identification and response are a particularly important safety aspect requiring careful consideration. The relational qualities of GenAI chatbots mean that users may disclose suicidal ideation or severe distress more freely than they would to a human, raising the stakes of an inadequate response considerably. Yet crisis protocols in this review were inconsistent, with some studies providing only static signposting to external resources. Several studies did not include crisis identification modalities in the first place. Crisis management should be treated as a fundamental safety requirement with clearly outlined identification, escalation and response protocols. No consensus standards for crisis identification in GenAI models currently exist, but benchmarking efforts that evaluate chatbot responses to simulated crisis scenarios represent emerging steps toward establishing minimum safety requirements [50]. Established regulatory frameworks such as the UK Medicines and Healthcare products Regulatory Agency (UK MHRA) guidance on digital mental health technologies provide safety and adverse event reporting standards for DMHIs, but require adaptation to address the unique challenges of autonomous AI-mediated interactions [53]. For such interactions, human-in-the-loop protocols, where clinicians or trained staff review conversations to detect harmful output and user expressions of distress represent an ideal crisis management approach. However, intervention designs requiring manual review of every interaction, as described by Heinz et al. [16], are unlikely to scale in real-world settings. More feasible alternatives could include layered content filtering and risk classifier models that triage conversations, flag elevated risk for targeted human review, and log safety events to enable continuous system improvement. Such approaches may better balance scalability with safety considerations. Collectively, these review findings reinforce the view that safety in GenAI mental health interventions extends beyond algorithmic safeguards but depends on coordinated technical, procedural, and governance mechanisms across the intervention lifecycle.

The evidence base is limited by inconsistent and inadequate safety reporting. No study employed standardised or validated instruments to capture GenAI-specific harms such as hallucinations, crisis response failures, or algorithmic bias. Importantly, adverse events were rarely tracked across the included studies and as such, crises and safety failures most likely went undetected. Reporting of GenAI system characteristics was similarly inconsistent, with many studies providing minimal detail on model architecture, training data, or technical safety evaluations, which limits reproducibility and independent risk assessment. Equity considerations were also largely absent, despite well-established evidence that large language models encode social biases related to gender, race, and sexuality [14,54,55]. Only a small number of included studies reported culturally adapting their interventions or evaluating performance across diverse populations. Without such evaluation, biassed or culturally inappropriate chatbot responses may go undetected, potentially compounding existing disparities in mental healthcare access and quality. These gaps point to the absence of a shared methodological and reporting framework for safety in this field. Future work would benefit from the development and adoption of standardised safety taxonomies, prospective adverse event monitoring protocols, and transparent reporting guidelines tailored to GenAI mental health interventions. Research efforts in response to these needs are ongoing, with benchmarking platforms such as MindBench AI enabling the systematic evaluation of technical features, clinical reasoning, and privacy protections across Gen AI mental health tools [56]. Clinically grounded AI safety frameworks such as VERA-MH also specifically allow for the evaluation of crisis handling and suicide risk detection across Gen AI mental health interventions [57]. Such model-level benchmarks are an important start but alone are insufficient. Broader attention to content management systems, structured workflows, safety layer architectures, and crisis response protocols is still needed to ensure user safety and inform regulatory policy. Although no universally accepted crisis response standard exists specifically for GenAI mental health chatbots, emerging guidance from the World Health Organization and the UK government converges on several minimum safety expectations. These include the ability to detect crisis signals (e.g., suicidality or psychosis); provide tiered risk-sensitive responses; avoid harmful or dependency-promoting interactions; and escalate high-risk users to human or emergency support through clearly defined crisis protocols [58,59].

This review’s findings have several implications for the design, evaluation, and governance of GenAI mental health chatbots. First, there is a need for more rigorous evaluation including efficacy trials that compare generative systems not only to wait-list control conditions, but also to active controls including rule-based chatbots and established low-intensity interventions. Such studies should be adequately powered, include diverse populations, and extend follow-up to capture both benefits and harms over time. Second, evaluation frameworks should move beyond symptom scales to include new instruments that can capture GenAI-specific safety outcomes. This aligns with the US FDA Digital Health Advisory Committee’s recommendations on GenAI-enabled mental health applications, which emphasised the need for post-market safety monitoring, AI-specific adverse event definitions, and human escalation pathways for crisis situations [22]. Harmonised benchmarks and standardised reporting checklists for conversational AI in mental health could also help improve transparency about model architecture, training data, safety guardrails, and crisis protocols [60]. Third, data privacy and regulatory oversight are essential to ongoing safety. Users often disclose highly sensitive information to chatbots; as such, transparent privacy policies, informed consent procedures and secure data management are required. As GenAI systems are increasingly deployed as therapeutic tools rather than wellness apps, regulators should consider them high-risk digital health technologies requiring ongoing oversight [50,61]. This includes independent testing, continuous performance and safety monitoring, and alignment with Software as a Medical Device (SaMD) and health AI regulations [22,60]. Finally, the development process itself is an important consideration for contextual relevance and safety. Interventions that engage mental health experts and people with lived experience throughout design and testing are more likely to align chatbot behaviour with the local context and user preferences. Meaningful co-design with end users, including those from marginalised and culturally diverse populations, can support inclusive and culturally responsive GenAI interventions [4,50,62].

### Limitations

The rapid pace of innovation in this field means that some included systems may have been updated or discontinued since publication, and newer architectures not represented in this review may carry different safety profiles. The exclusion of non-English language studies and the under-representation of proprietary commercial systems limit the generalisability of findings, as commercial developers may implement safety architectures that are not described in peer-reviewed literature. Heterogeneous study designs, variable safety reporting, and sparse adverse event documentation also constrain comparability across interventions. These constraints reflect the emerging nature of the field and highlight the need for more rigorous, transparent, and standardised safety evaluation.

## 5. Conclusions

This review offers a foundation for safer design and deployment of GenAI mental health chatbot interventions. The evidence shows that responsible deployment is achievable through deliberate integration of layered technical safeguards, human oversight, structured crisis protocols, and meaningful user co-design. Advancing the field will require shared standards for pre-deployment testing, systematic adverse event reporting, and regulatory oversight proportional to the clinical risks these systems carry. Without these considerations, purpose-built interventions risk failing to deliver meaningful safety advantages over the general-purpose chatbots they were designed to improve upon.

## Figures and Tables

**Table 1:** Study Characteristics and Features of Generative AI Mental Health Chatbot Interventions.

Study ID (Chatbot Name)	Population	Study Design and Outcomes	Therapeutic Approach and Delivery Modality	Conversational Agent Features	Underlying AI Technique and Safety Mechanism
Campellone 2025 (Woebot) [29]	General adult users with mood or anxiety symptoms (USA)	2-week double-blind exploratory RCT comparing generative vs rule-based versions. User satisfaction, engagement, working alliance, and safety (technical guardrails and adverse events).	Guided CBT-based digital self-help for mood and anxiety; psychoeducation and self-management tools. Participants used the chatbot daily for two weeks as part of a clinical trial.	Empathy engine detecting and classifying user mood; dynamically tailored activities. Mood tracking, journaling, educational modules.	OpenAI Ada-002 and davinci-003 LLMs fine-tuned on Woebot proprietary user-conversation and FAQ datasets. Proprietary NLP classifier for detecting concerning language. RAG via content management system retrieving from curated psychoeducational library. Ensemble of classification models filtering hate, sexual violence, and self-harm language as output validation step.
Espinoza 2023 (Ana) [30]	Dementia caregivers (Peru)	Mixed-methods design: stakeholder engagement, chatbot development, user evaluation. Feasibility, satisfaction, generative AI vs structured chatbot comparison.	Evidence-based conversational strategies (BATHE method) for dementia caregiver emotional and informational support. Web-based, On-demand delivery.	Chatbot facilitates caregiving reflections; provides psychoeducation on behavioural symptoms; crisis-alert and health-navigation features. Has a “Vent” feature for emotional release.	GPT-3.5 enhanced with RAG from expert-verified dementia resources via semantic similarity matching. Fine-tuned on caregiver-support literature and expert-curated dementia Q&A. Prompting for personalisation and guiding expansion of retrieved content. Thresholding mechanism retrieving verified content when available.
Guo 2024 (HopeBot) [31]	Simulated user personas for depression screening (UK)	Mixed-methods development and preliminary evaluation with simulated users. Feasibility, acceptability, and satisfaction with GPT-based voice chatbot.	Empathetic interaction and personalised support for depression screening using PHQ-9 logic. Deployed via mobile app, with voice interface. Conducted as single-session simulated dialogues using 10 diverse personas.	Speech-to-text and natural conversation to explain screening questions, offer empathetic clarifications, and provide general emotional support post-screening.	GPT-3.5-Turbo integrated with Whisper for speech recognition and custom prompt logic. Structured prompts enforcing PHQ-9 question order, scoring rules, and discouraging deviation from screening script. No fine-tuning reported.
Habicht 2024 (Limbic Care) [32]	Adult NHS patients in group CBT (UK)	Multisite real-world quasi-experimental study. Engagement, adherence, treatment success vs workbook control; user satisfaction and perceived benefits.	Therapeutic support tool integrated into ongoing group CBT; helps users work through clinician-assigned exercises. Mobile app-based intervention used between sessions for 8–10 weeks to enhance engagement and treatment adherence.	Limbic Layer for real-time emotion recognition and tailored feedback. Session summaries, progress visualisation, and check-ins.	GPT-4 LLM augmented by proprietary emotion-recognition and ML modules (Limbic Layer) for clinical decision-making and safety. Trained on anonymised clinical CBT dialogue from NHS therapy sessions. Clinicians monitored conversations for harmful responses and regulatory compliance.
Heinz 2025 (Therabot) [16]	Adults with major depression, anxiety disorders, and eating disorders (USA)	Waitlisted RCT. Symptom reduction (PHQ-9, GAD-7, CHR-FED), usability, and safety at 4- and 8-week follow-up.	Structured third-wave CBT intervention for depression anxiety and eating disorders, delivered over 4 weeks with daily conversational sessions. Designed for self-management with periodic follow-up.	Multi-thread conversation interface, contextual memory, safety guardrails, scheduled notifications. Generates empathic reflections, goal setting, and reinforcement of therapeutic progress.	LLM ensemble (Falcon-7B + LLaMA-2-70B) fine-tuned via QLoRA on therapist-patient dialogue incorporating third-wave CBT transcripts. Crisis detection model screening messages for crisis-related language. Prompt engineering for evidence-based strategies. Rigorous human-in-the-loop (HITL): all responses supervised by clinicians, who intervened on 13 occasions for inappropriate content.
Javanbakht 2024 (ExpandXR) [33]	First responders with PTSD (USA)	Qualitative user experience study with clinician observations. Feasibility, user engagement, emotional response, and real-world functional improvements.	AI-enhanced augmented reality exposure therapy (ARET) for PTSD and anxiety. Deployed via AR headsets(Microsoft HoloLens, Apple Vision Pro) in therapist-supervised 30-60 min sessions over multiple weeks.	Holographic AI-human avatars with realistic unscripted conversations. Tracks physiological response and adjusts difficulty in real time. Customisable scenarios and sound effects controlled by clinician.	GPT-4-driven dialogue system embedded within ARET environment. Prompt engineering for scenario customisation and persona definition. Fine-tuned on simulated clinician-patient exposure therapy transcripts. Clinicians observed AI character behaviour and could adjust exposure intensity or stop the scenario.
Lai 2023 (Psy-LLM) [34]	General population and mental health professionals (Australia)	Prototype development and evaluation study. Conversational quality, model coherence, fluency, and relevance.	LLM-assisted professional support tool offering screening, triage, psychoeducation, and CBT-based support. Deployed via Web platform, with 24/7 on-demand access.	Dual-purpose real-time automated Q&A assistant. Generates recommendations for health professionals and acts as standalone tool for patients when no counsellors are available.	Large LLMs (200B parameter PanGu by Huawei + 3.5B WenZhong by Idea Research Institute) fine-tuned on PsyQA and Chinese counselling dataset.
Lin 2023 (Unnamed) [35]	Office workers (China)	Pre-post test experimental design. Well-being and life-satisfaction improvement after chatbot use: usability and acceptability.	Two-week stress management combining empathy theory and positive psychology through brief daily sessions.	Open-ended empathetic conversations; validates emotions and guides users through relaxation and thought-challenging techniques.	RoBERTa-wwm-ext-large for emotion and cognitive distortion recognition. CDial-GPT (GPT-2 architecture Chinese LLM) for empathy-based natural language generation. Trained on proprietary annotated dialogue data from mental health practitioners.
Liu 2024 (ComPeer) [36]	University students experiencing significant stress (China)	Mixed-methods pre-post quasi-experimental design with qualitative user feedback. Stress reduction, UX, and satisfaction.	Peer-support intervention with proactive check-ins and stress management. Daily use for one week. Web-based delivery via Tencent QQ messaging platform.	Customisable peer support AI persona with event detection and scheduling. Anticipates user stress points; initiates supportive messages and encourages self-reflection.	GPT-4-based CA with memory and scheduling modules. Chain-of-Thought (CoT) prompting combined with memory module to encourage context-aware responses and reduce hallucinations. Iterative participant feedback incorporated into design refinements.
Manole 2024 (Unnamed) [37]	Adults with mild-moderate anxiety (Romania)	Two-phase observational study (short- and longer-term follow-up). Anxiety score reduction; engagement metrics (frequency and duration of interactions).	Brief CBT-based intervention for stress and anxiety via daily self-paced dialogues for one week. Combines behavioural activation, mindfulness, and breathing exercises.	Analyses text input to identify emotional cues and symptom patterns. Rule-based system integrated with CBT principles to guide user through suitable intervention. Adaptive learning for intervention tailoring.	ChatGPT-based LLM. Prompt engineering grounded in clinically validated assessment tool (GAD-7 structure). NLP-driven symptom analysis. RLHF loop allowing users to confirm identified emotional classifications to avoid misinterpretations.
Nazarova 2023 (TeaBot) [38]	University students (Kyrgyzstan)	RCT. Reduction in cognitive distortions; user engagement metrics.	CBT-based self-guided psychoeducation and cognitive restructuring using Socratic dialogue. Deployed via telegram chatbot as an eight-week intervention with weekly minimum engagement.	CA detects cognitive distortions in user input; uses Socratic questioning to challenge maladaptive thoughts; provides feedback summaries.	GPT-3 (Curie for cognitive distortion classification, Davinci for response generation). RAG augmenting Socratic questioning. Trained on 240 cognitive distortion examples curated from CBT literature and validated by psychologists. Uncertainty response allowing model to abstain when classification confidence was low.
Ng 2023 (CareBot) [39]	Working adults with occupational stress (Malaysia)	Mixed-methods ablation study. Stress reduction, usability of emotion-aware CBT chatbot; impact of emotion detection and cultural adaptation on UX.	CBT-based intervention for occupational stress, culturally adapted to Malaysian context via language and content. Delivered via Slack app platform.	Emotion detection integrated with culturally adapted CBT modules; tailored exercises and visual feedback on progress.	Fine-tuned BERT for emotion detection integrated with GPT-3.5 for response generation. Fine-tuned on five public emotion-recognition datasets (SemEval-2018, DailyDialogue, MELD, etc.). Cultural and linguistic adaptation of training data and content.
Ogamba 2023 (Wellness Buddy) [40]	University students (Kenya)	Conversational agent design and deployment architecture study. Feasibility and design description of culturally relevant wellness chatbot.	CBT-based psychoeducation and self-guided reflection for wellbeing promotion and mental health literacy. Deployed via mobile app for on-demand self-guided use.	Psychoeducational chatbot with reflective prompts, mood check-ins, brief self-help exercises, and referral information to professional support. Learning centre with verified resources.	Fine-tuned LLM on mental health conversational data from Kaggle, augmented with synthetic data to cover more user intents. Local data storage proposed for privacy. Non-adapted publicly available datasets used.
Sabour 2023 (Emohaa) [41]	Adults with depression and anxiety (China)	Three-arm RCT (Emohaa vs rule-based chatbot vs waitlist). Symptom reduction in depression, anxiety, and insomnia (ISI); acceptability and user satisfaction.	CBT-based self-help with guided expressive writing (ES-Bot) and structured CBT exercises (CBT-Bot). Delivered via WeChat platform over eight weeks with recommended daily engagement.	Two CAs: CBT-Bot (rules-based) and ES-Bot (hybrid rules + LLM). ES-Bot allows open-ended emotional conversations; tracks engagement, mood, and adherence.	EVA2.0 pretrained Chinese dialogue model (2.8B parameters). Additional model for suicide risk classification. Fine-tuned on Chinese emotional-support datasets (ESConv) and CBT dialogue data. Human moderators monitored conversations and reviewed flagged content.
Schäfer 2025 (Clare R) [42]	English-speaking self-referred adults with anxiety, depression, and loneliness (Germany)	Cross-sectional survey within a longitudinal evaluation. User characteristics, motives, expectations, and therapeutic alliance with multimodal GenAI chatbot.	CBT and mindfulness-informed intervention with Socratic dialogue, cognitive restructuring, and guided reflection. Users accessed platform asynchronously on demand but needed to complete at least one interaction between measurement periods (4-week intervals) to be eligible for study.	Empathetic voice and text dialogue with adaptive tone modulation. Assesses user mental state and suggests relevant exercises. Alternatives offered if exercises declined.	Hybrid multi-LLM fine-tuned on therapist-client dialogue and expert scripts. NLP to process voice transcriptions. Rules-based system developed by psychologists combined with generative AI to match user input to relevant exercises. Separate ML model filtering both inputs and outputs, over-flagging potentially harmful content for human review. Human moderators reviewed flagged content.
van der Schyff 2023 (Leora) [43]	Not specified (Australia)	Development study. Platform feasibility and ethical considerations for Leora chatbot.	Mental health first aid techniques grounded in CBT and ACT. Symptom screening, brief motivational dialogue, and referrals. On-demand access via Web and mobile app.	Self-guided digital coach for mental health assessment and resource linkage. Offers symptom screening, brief motivational dialogue, and referrals to human support.	NLP with humanistic dialogue framework. Rigorous privacy architecture including data encryption and compliance with national privacy law. Users granted rights to access and delete data. Crisis protocols blocked further app use when safety flagged.
Vossen 2024 (Personal AI assistant) [44]	Dutch-speaking adults (Belgium)	RCT with active control. Impact of personalisation on therapeutic bond and usage intentions.	Personalised psychotherapy chatbot for general mental health support. Single-session CBT-based intervention offering a choice of Socratic style (using open-ended questions) or a goal-oriented style (giving concrete advice).	“AI therapist” that adapts conversational tone and content to user personality in real time to strengthen therapeutic bond. Features customizable avatar, personality and therapy style. Also has an editable “brain box” that stores learned traits of the user.	ChatGPT API for lower-risk, supportive conversation. Rasa for intent recognition and detection of high-risk topics. When high-risk topics detected, ChatGPT bypassed entirely and rule-based responses signposting crisis lines served. Co-design with students and medical experts before broader testing.
Wang 2024 (ArtTheraCat) [45]	University students with exam-related stress (China)	Mixed-methods pre-post quasi-experimental design with semi-structured interviews. Positive-affect improvement; usability of multimodal (text + image) chatbot.	Art therapy-based emotional support using text dialogue combined with AI-generated imagery. Three intervention phases included emotional visualisation and externalisation through image generation, art-based storytelling, and positive reinforcement. Delivered via single sessions lasting 30-40 min.	Avatar-based multimodal design integrating animated visuals and art generation. Prompts instructed model to act as supportive art therapist, manage conversation flow, track image history.	Generative image model integrated with LLM dialogue. Prompt engineering instructing model to act as supportive art therapist, avoid creating distressing images, manage conversation flow, and track image history.
Xygkou 2024 (MindTalker) [46]	People with early-stage dementia and caregivers (UK)	Two-phase iterative co-design + user evaluation. User perceptions of companionship, empathy, and ethical challenges of AI agent.	AI-enhanced audio chatbot for conversational reminiscence therapy promoting social engagement. Based on psychological principles of emotional support, grounding techniques, structured routine, and behavioural reinforcement.	Voice interface with speech-to-text. Photo-based reminiscence therapy for early dementia.	GPT-4 with prompts to align responses with reminiscence therapy. Iterative co-design with experts and end-users resulting in final version selected after eleven iterations based on UX and safety considerations.
Yu 2024 (DrBot) [47]	University staff and students (UK)	Experimental model-comparison study. Conversational quality and response accuracy across ChatGPT-3.5, DialoGPT, and hybrid models.	Cognitive Behavioural Therapy (CBT) principles for psychological support. Delivered as a web-based single-session intervention for mental health support.	Gamified avatar interaction with “calming” pictorial characters representing the user and a psychologist. Users start the “game” to initiate conversation with the psychologist	Hybrid fine-tuned DialoGPT + ChatGPT 3.5. Prompt engineering to refine responses and limit harmful content. Mental health professionals evaluated DrBot during development.
Zhang 2024 (VCounselor) [48]	Individuals with subclinical distress (China)	Experimental model-comparison study. Satisfaction, engagement, trust, and perceived effectiveness of knowledge-enhanced LLM intervention.	Psychological counselling chatbot tested in a one-session experiment. Designed to simulate counselling conversations and assess user satisfaction with knowledge-enhanced interactions.	Knowledge retrieval system mapping user utterances to DSM-5 diagnostic frameworks. Avatar-based multimodal design with animated visuals. Leverages memory module to adjust conversational style.	LLM (ChatGLM2-6B) augmented by RAG pipeline incorporating structured DSM-5 data. User utterances mapped to diagnostic frameworks and relevant knowledge snippets retrieved to guide LLM responses. Knowledge-enhanced approach to reduce hallucination and ensure clinical plausibility.

## Data Availability

No new data were created or analysed in this study.

## Supplementary Materials

The following supporting information can be downloaded at: https://www.mdpi.com/article/10.3390/healthcare14101395/s1, PRISMA-ScR reporting checklist.

## Appendix A

**Table A1:** Full Search Strategy for MEDLINE/PubMed. Ovid MEDLINE(R) ALL <1946 to July 2025>.

	Search Terms	Hits
1	exp artificial intelligence/or exp natural language processing/or exp neural networks, computer/	243,593
2	(“Artificial Intelligence” or “Generative Artificial Intelligence” or “Generative AI*” or “Conversational Artificial Intelligence” or “Conversational AI*” or “Conversational Agent*” or “Conversational bot*” or “Conversational system*” or “AI Chatbot*” or “AI-Chatbot*” or “Chatbot*” or “Chat bot*” or “Chat-bot*” or “Relational agent*” or “Virtual agent*” or “Virtual coach*” or “Virtual Assistant*” or “Digital assistant*” or “Virtual therap*” or “virtual human*” or “AI-based*” or “AI based*” or “AI driven*” or “AI-driven” or “AI-enhanced*” or “AI enhanced*” or “AI-enabled” or “AI enabled” or “AI-powered” or “AI powered” or “Natural Language Processing” or “NLP or Large Language Model* or LLM* or Generative Pre?trained Transformer* or GPT* or ChatGPT* or Chat GPT*”).mp.	123,238
3	1 or 2	295,187
4	exp psychotherapy/or exp behavior therapy/or exp psychosocial intervention/	232,911
5	(“psychological intervention*” or “psychological therap*” or “psychological treatment*” or “psychosocial intervention*” or “psychosocial therap*” or “Behavio?ral therap*” or “cognitive behavio?ral therap*” or “CBT” or “iCBT” or “CBT-based” or “mental health intervention*” or “mental support” or “emotional support” or “Digital mental health” or “digital mental health intervention*” or “DMHI” or “e-mental health*” or “mental health app*” or “mental wellness app*” or “mental wellbeing app*” or “mental health platform*” or “online mental health*” or “telepsychiatry” or “digital psychotherap*” or “digital psychiatry”).mp.	91,410
6	4 or 5	281,264
7	exp Mental Disorders/	1,556,968
8	exp Substance-Related Disorders/	328,448
9	(anxiety or “anxiety disorder*” or depress* or bipolar* or “mood disorder” or phobia* or psychosis or psychotic* or “psychological stress” or “psychological distress” or “post-traumatic stress disorder*” or PTSD or “self-harm” or “self harm” or suicid* or “substance use*” or “substance abuse” or “drug abuse” or “alcohol abuse” or “alcohol misuse” or “mental disorder*” or “mental illness*”).mp.	1,462,098
10	7 or 8 or 9	2,381,780
11	3 and 6 and 10	635
